# *ZmARF16* Regulates *ZCN12* to Promote the Accumulation of Florigen and Accelerate Flowering

**DOI:** 10.3390/ijms25179607

**Published:** 2024-09-05

**Authors:** Zhenzhong Jiang, Yang Zhao, Bai Gao, Xiaotong Wei, Peng Jiao, Honglin Zhang, Siyan Liu, Shuyan Guan, Yiyong Ma

**Affiliations:** 1 College of Life Sciences, Jilin Agricultural University, Changchun 130118, China; 18722126836@163.com; 2 Joint International Research Laboratory of Modern Agricultural Technology, Ministry of Education, Changchun 130118, China; 18943278176@189.cn (Y.Z.); gaobai20220632@163.com (B.G.); wxt5727496910@126.com (X.W.); m18404319202_1@163.com (P.J.); 18722186836@189.cn (H.Z.); siyanliu@sohu.com (S.L.); 3 College of Agronomy, Jilin Agricultural University, Changchun 130118, China

**Keywords:** *ZmARF16*, *ZCN12*, flowering, auxin, florigen

## Abstract

Auxin response factors(ARFs) are a class of transcription factors that regulate the expression of auxin response genes and play a crucial role in plant growth and development. Florigen plays a crucial role in the process of flowering. However, the process by which auxin regulates the accumulation of florigen remains largely unclear. This study found that the expression of *ZmARF16* in maize increases during flowering, and the genetic transformation of *ZmARF16* accelerates the flowering process in *Arabidopsis* and *maize*. Furthermore, *ZmARF16* was found to be positively correlated with the transcription of the *ZCN12* gene. Similarly, the FT-like gene *ZCN12* in *maize* rescues the late flowering phenotype of the FT mutation in Arabidopsis. Moreover, *ZCN12* actively participates in the accumulation of florigen and the flowering process. Further research revealed that *ZmARF16* positively responds to the auxin signal, and that the interaction between ZmARF16 and the *ZCN12* promoter, as well as the subsequent promotion of *ZCN12* gene expression, leads to early flowering. This was confirmed through a yeast one-hybrid and dual-luciferase assay. Therefore, the study provides evidence that the *ZmARF16*-*ZCN12* module plays a crucial role in regulating the flowering process of *maize*.

## 1. Introduction

Maize is a crucial food crop around the world, originating from the tropical Mexican region, and later domesticated and rapidly expanding to temperate regions. The flowering traits of corn were subject to artificial selection during the domestication process, but the genetic basis of this process is still not well understood. Higher plants undergo a series of developmental stages, including seed germination, nutritional growth, flowering, fertilization, embryonic development, and seed formation, with flowering being an important marker of the transition from nutritional growth to reproductive growth [1]. Flowering locus T (FT) is a widely studied flowering integrator, with its protein synthesized in leaves and transported to the apical meristematic tissues, where it interacts with other proteins to promote flowering [2]. *FT* and *Terminal Flower1(TFL1)* are important genes downstream of the flowering regulation network, belonging to the phosphatidylethanolamine-binding protein (PEBP) family, with a conserved PEBP domain and 70% amino acid similarity [3]. *FT* and *TFL1* play opposite roles in regulating plant flowering, with *FT* promoting flowering and *TFL1* inhibiting flowering. This functional switch is caused by a single critical amino acid residue and a conserved amino acid fragment in the PEBP domain [4]. The critical amino acid residue in the PEBP domain is tyrosine at position 85 in FT and cysteine at position 88 in TFL1, and exchanging these residues can result in a functional switch of the two proteins. In addition, the amino acid positions 140 glutamine (Gln, Q) and 144 aspartic acid (Asp, D) also play a crucial role in determining the function of the FT and TFL1 genes, with position 140 being Q in FT and D in TFL1. The amino acids tryptophan (Trp, W) at position 138 and asparagine (Asn, N) at position 152 in the FT gene are relatively conserved and essential [5].

Auxin was the first class of plant hormone to be discovered by people, and subsequent extensive research has revealed that auxin plays a critical role in regulating various processes in plant growth and development [6]. Auxin response factors (ARFs) are a unique class of transcription factors in plants that regulate the transcription of downstream target genes by specifically binding to auxin response elements (Aux REs). Research has found that the expression levels of most ARF genes are significantly higher in reproductive organs than in nutritional organs [7]. The auxin signaling process involves early-response genes (such as AUX/IAA, GH3, and SAUR gene families) and ARF family genes that interact with Aux REs. ARF proteins bind to the auxin core response elements (5′-TGTC-3′ or 5′-TGTCTC-3′) of auxin-regulated gene promoter regions, inhibiting or activating the transcription of these genes (8).

The ARF gene family is an important transcription factor family in plants. Studies have shown that ARF proteins contain three conserved domains, including the plant-specific DNA-binding domain B3 (DBD) located at the NH2 terminus, the activation or inhibition structure (Auxin-resp domain) in the middle region (MR), and the dimerization domain (PB1) at the COOH terminus [8]. The B3 domain requires amino acid assistance to bind to the cis-acting element AuxRE, and the binding is not related to the use of exogenous auxin. The PB1 domain is more conserved and has a high degree of similarity to the Aux/IAAs domain, which can produce a dimerization region. For the middle region, if the amino acid sequence contains a large amount of glutamine, serine, and leucine, this region has an activating effect, and if these amino acids are replaced by proline, glycine, or other residues, this region has an inhibitory effect [9]. To date, 23 ARF transcription factors have been identified in the genome of the model plant Arabidopsis, and the functions of most members have been verified [10]. The analysis results show that *AtARF1* and *AtARF2* have some common functions and may positively regulate the occurrence of plant leaf senescence and organ abscission [11]. Studies have found that *AtARF3* is a necessary functional complex for membrane development and leaf polarity establishment in *Arabidopsis*, which depends on auxin regulation [12]. Zhang et al. found that *AtARF6* and *AtARF8* play important roles in regulating the development of stamens and pistils, fruit development, fertilization, anther development, and pollen formation in *Arabidopsis* [13]. *AtARF7* and *AtARF19* transcription factors directly or indirectly mediate lateral root formation in Arabidopsis [14].

In this study, the *ZCN12* gene of the PEBP family was found to be highly expressed during the flowering period of *maize*, and the expression of the *ZmARF16* gene increased. To explore whether *ZmARF16* is involved in regulating *ZCN12* to promote flowering, yeast one-hybrid and dual-fluorescence experiments were used to demonstrate that ZmARF16 interacts with the *ZCN12* promoter to promote *ZCN12* gene expression. Transgenic Arabidopsis plants showed that *ZCN12* promotes flowering. *ZmARF16* can respond positively to auxin signals, and fluorescence quantitative analysis showed that its expression increased during the flowering period. Thus, this study suggests that *ZmARF16* responds positively to auxin signals and promotes the expression of the *ZCN12* gene to promote *maize* flowering.

The wild type is the *maize* inbred line D12164, which was previously preserved in the laboratory (plant height—183 cm; flowering period—75 days). Early-flowering *maize* M12164 was obtained via the EMS mutagenesis of D12164 (plant height—180 cm; flowering period—68 days). The sequencing data were uploaded to NCBI.

## 2. Results

### 2.1. Analysis of the Gene Structure and Expression of ZmARF16 and ZCN12

We conducted evolutionary analyses between the ARF family genes in *maize* and those in Arabidopsis, and found that ZmARF9, ZmARF16, ZmARF18, and ZmARF22 in *maize* have similar evolutionary relationships with AtARF6 and AtARF8 in Arabidopsis. Subsequently, fluorescence quantitative analysis was conducted during the flowering period of *maize*. Finally, it was found that the expression level of ZmARF16 was the most significant (Figure S1A), so ZmARF16 was chosen as the research object.

**Figure 1:**
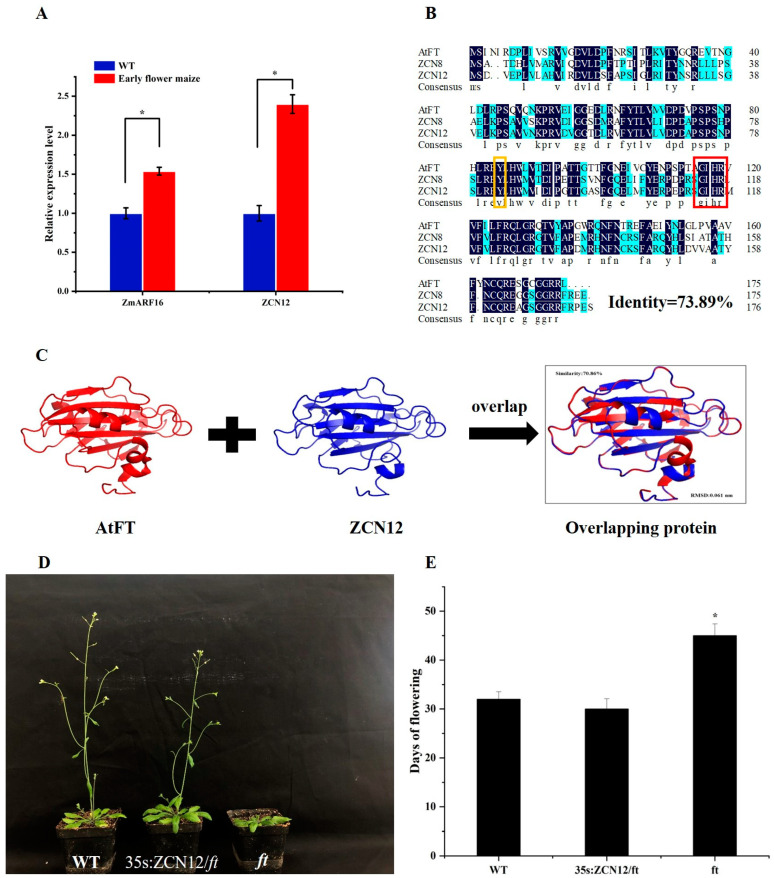
Expression patterns, homologous proteins, sequence alignment, and functional analysis of *ZCN12* gene. (**A**) Expression pattern analysis of *ZmARF16* and *ZCN12* genes during flowering period in an early-flowering mutant. (**B**) Amino acid sequence alignment of *AtFT*, *ZCN8*, and *ZCN12*. Yellow frame—position 85 amino acid; red frame—conserved GxHR domain. (**C**) Three-dimensional structure alignment of ZCN12 and AtFT proteins. (**D**) Phenotypes of WT, 35s:ZCN12, and FT lines under a day-long photoperiod. (**E**) Flowering times of WT, 35s:ZCN12, and FT lines under day-long photoperiod. * *p* < 0.05.

By analyzing the transcriptome sequencing of different developmental stages of early-flowering *maize*, we found that ZmARF16 was highly expressed during the flowering stage, and the expression of the ZCN12 gene was also high (Figure 1A). We speculated that ZmARF16 may be related to *maize* flowering. Subsequently, based on the analysis of the ZmARF16 sequence from the NCBI database (https://www.ncbi.nlm.nih.gov/protein/NP_001333732.1 (accessed on 1 May 2023)), it was found that the conserved regions mainly include three structural domains, namely the DNA-binding domain (DBD), the middle region (MR), and protein-binding domain 1 (PB1), and these are most closely related to AtARF6 in terms of evolution (Figure S1B,C). Then, a comparison of the amino acid sequences of ZCN12, ZCN8, and AtFT was performed, and the sequence homology was found to be 73.89%. The 138th amino acid AtFT is W, while ZCN8 and ZCN12 are M. This may be due to the distant blood relationship between Arabidopsis and *maize*, but ZCN8 has been validated to regulate *maize* flowering, and its important position is similar to that of AtFT. The amino acids at positions 140 and 152, although slightly different, show high homology and similarity. Therefore, ZCN12 has high similarity and homology in amino acid sequences with AtFT and ZCN8 (Figure 1B). A comparison of the tertiary protein structures of ZCN12 and AtFT was also performed, and the Root Mean Square Deviation(RMSD) was found to be 0.061 nm (the smaller the RMSD, the more similar the protein structure), while the protein similarity reached 70.86% (Figure 1C). Based on the key amino acid sites, ZCN12 was found to be an FT-like gene with a positive effect on flowering.

### 2.2. ZCN12 Can Rescue the Late Flowering Phenotype of the Arabidopsis FT Mutant

Through the analysis of ZCN12′s expression pattern, a comparison of ZCN12 and AtFT amino acid sequences, and a comparison of their protein tertiary structures, we found that ZCN12 and AtFT have high similarity. In order to investigate the impact of ZCN12 on flowering, we then constructed FT *Arabidopsis* plants and overexpressed the ZCN12 gene in FT *Arabidopsis*. The study showed that the flowering time of *Arabidopsis* was delayed after FT was lost, but the flowering time in the 35S:ZCN12/ft *Arabidopsis* plant was capable of being restored to normal levels (Figure 1D,E). This indicates that ZCN12 can rescue the late-flowering phenotype related to FT mutation in *Arabidopsis*, and has a positive effect on flowering.

### 2.3. Identification of the ZmARF16 and ZCN12 Gene

**Figure 2:**
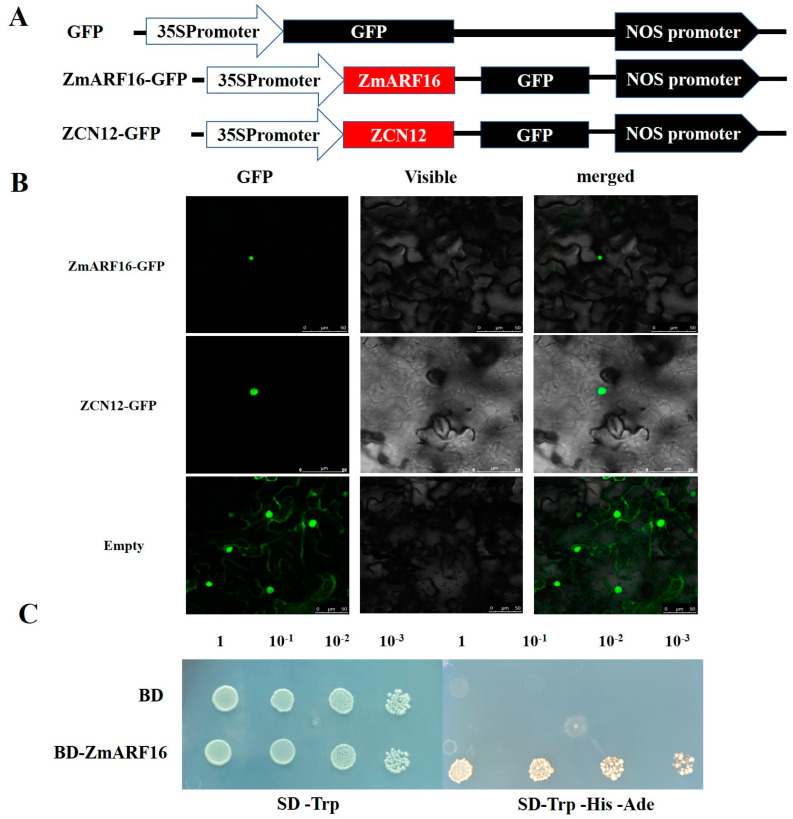
*ZmARF16* transcription factor and *ZCN12* characteristics. (**A**) Structural diagrams of the pCAMBIA1302–GFP vector, pCAMBIA1302-ZmARF16–GFP construct, and pCAMBIA1302–ZCN12–GFP construct. (**B**). Subcellular localization of ZmARF16 and ZCN12 in tobacco leaf mesophyll cells. Scale bar = 50 μm. (**C**) Transcriptional activity of *ZmARF16*.

The amino acid sequence analyses of ZmARF16 and ZCN12 predicted their localizations in the nucleus. ZmARF16-GFP and ZCN12-GFP vectors were constructed, and their expressions in tobacco leaves validated this hypothesis. This study found that the green fluorescent signals of ZmARF16-GFP and ZCN12-GFP were only detected in the nucleus, whereas only GFP green fluorescence was observed throughout the entire cell (Figure 2), indicating that ZmARF16 and ZCN12 specifically aggregate in the nucleus.

To investigate the transcriptional activation activity of ZmARF16, we fused its coding sequence (CDS) with the GAL4 DNA-binding domain (DBD) to create the pGBDT7-ZmARF16 construct (BD-ZmARF16). Subsequently, BD-ZmARF16 and the empty vector pGBDT7 (BD) were transformed into yeast as negative controls for transcriptional activity analysis. As depicted in Figure 2C, yeast strains carrying BD-ZmARF16 exhibited robust growth on SD/–Trp–His–Ade media, whereas yeast strains harboring BD only grew on SD/-Trp media. This indicates that ZmARF16 functions as a transcriptional activator.

### 2.4. ZmARF16 Can Promote Flowering Time in Arabidopsis and Maize

**Figure 3:**
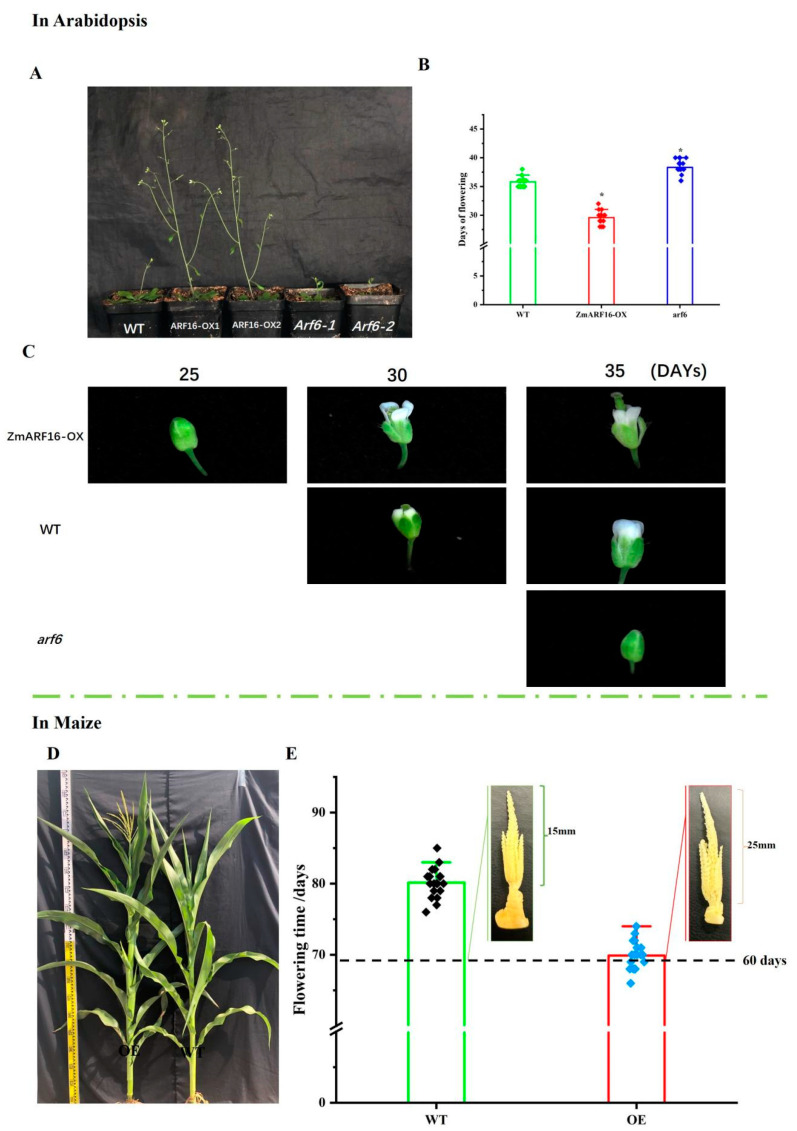
*ZmARF16* promotes the process of flowering. (**A**). Flowering phenotypes of WT, *ZmARF16*–OX, and arf6 lines. (**B**) Flowering times of WT, *ZmARF16*–OX, and *Atarf6* plants under a day-long photoperiod. (**C**) Flower development phenotypes of WT, ZmARF16–OX, and arf6 lines at 25, 30, and 35 days of growth. (**D**) The flowering phenotype of the *ZmARF16*–OX line. All tassels of *maize* were extracted. (**E**). Flowering times of the WT- and *ZmARF16*–OX-line plants. The growth status of female ears in *maize* at 60 days. *n* = 20. *: *p* < 0.05.

*ZmARF16* is a key gene in the auxin signal transduction pathway, as auxin can regulate flowering and flower development in plants. Therefore, we will investigate whether *ZmARF16* can affect flower development and flowering time. We introduced the 35S:ZmARF16 vector into *Arabidopsis* using the floral dip method. The transgenic *Arabidopsis* plants were propagated through successive generations. In the T3 generation, two plants with typical phenotypes were selected and named ZmARF16–OX1 and ZmARF16–OX2 (Figure 3A). They originate from the same parent. Similarly, by measuring the flowering times of *ZmARF16*–OX/arf6 and arf6, we found that *ZmARF16* can alleviate the loss of *AtARF6* function and ensure normal flowering (Figure S2A,B). Here, we obtained two overexpressing plant lines (*ZmARF16*–OX1, *ZmARF16*–OX2) and two *AtARF6* mutant plant lines (*Atarf6*–*1*, *Atarf6*–*2*). We compared the flowering times and plant phenotypes of *ZmARF16*–OX1, *ZmARF16*–OX2, *Atarf6*–1, *Atarf6*–2, and WT. Here, we found that *ZmARF16* can participate in the regulation of flowering time, and the overexpression of *ZmARF16* can accelerate flowering, while the mutation of AtARF6 can delay flowering (Figure 3B–C). After subsequently overexpressing the *ZmARF16* gene in *maize*, we observed a shortened flowering period, with this overexpressing *maize* flowering 10 days earlier. Furthermore, when examining the tassel of *maize* grown for 60 days, we found an accelerated developmental process in the overexpressing maize tassel (Figure 3D,E).

We found that *ZmARF16* can affect the flower’s meristematic tissue, as indicated by the results of microscopic examination. In contrast, the central meristematic region of WT has enlarged cells, and the columnar head is well developed (Figure S3A). The central meristematic region of arf6 develops slowly, as does its pistil; the cells are tightly arranged, and the number of cells is significantly lower than that in WT (Figure S3B). At the same time, ZmARF16–OX matures and fruits, and its seed development is obvious (Figure S3C).

### 2.5. ZmARF16 Positively Responds to the Auxin Signal in Arabidopsis

**Figure 4:**
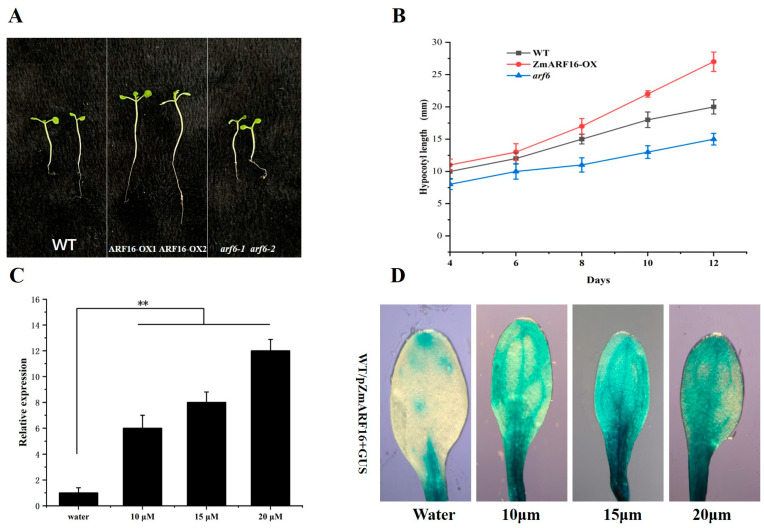
*ZmARF16* response to auxin signal. (**A**) Hypocotyl growth of WT, *ZmARF16*-OX, and *arf6* lines under 15 µM auxin treatment. (**B**) Hypocotyl elongation of WT, ZmARF16-OX, and arf6 lines every 4 days under 15 µM auxin treatment. (**C**). Expression analysis of GUS gene in pZmARF16:GUS plants under external water and 10 µM, 15 µM, and 20 µM auxin treatment. (**D**) GUS accumulation analysis in leaves of pZmARF16:GUS under external water and 10 µM, 15 µM, and 20 µM auxin treatment. ** *p* < 0.01.

ARF transcription factors regulate gene expression in response to different concentrations of auxin. These ARF transcription factors bind to auxin response elements in the downstream gene promoter and activate or repress gene transcription. To detect whether or not *ZmARF16* responds to auxin signals in vivo, we examined the growth response of the hypocotyl to auxin in *ZmARF16*–OX1, *ZmARF16*–OX2, *Atarf6*–1, and *Atarf6*–2. We added 15 µM auxin to the culture medium and observed that the hypocotyl of *ZmARF16*–OX1 and *ZmARF16*–OX2 elongated significantly compared to that of the wild type, while the growth of the hypocotyl in *Atarf6*–*1* and *Atarf6*–*2* was slow and elongation was limited (Figure 4A,B). We also applied water and 10 µM, 15 µM, and 20 µM of auxin to the leaves of pZmARF16:GUS plants and found that the gene expression of GUS was significantly upregulated (Figure 4C). The staining of pZmARF16:GUS plants showed that as the auxin concentration increased, more GUS accumulated, primarily in the veins of the leaves (Figure 4D). These results indicate that *ZmARF16* is a positive response factor for auxin, and that *ZmARF16* accumulates primarily in the veins of the leaves.

### 2.6. ZmARF16 Promotes the Accumulation of Florigen in Arabidopsis and Maize Protoplasts

**Figure 5:**
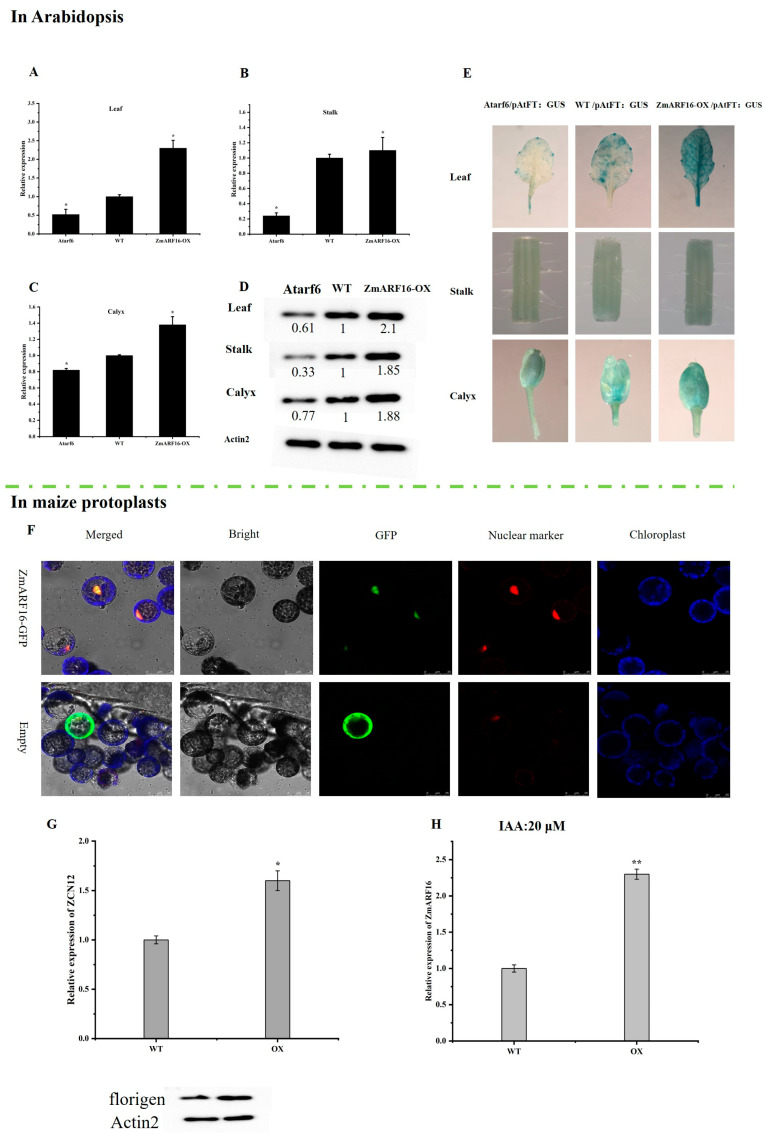
*ZmARF16* promotes florigen accumulation. (**A**–**C**). Expression levels of the FT gene in *ZmARF16*–OX, WT, and arf6 plants’ leaves, stems, and sepals. (**D**) Western blotting analysis of florigen in *ZmARF16*–OX, WT, and arf6 plants’ leaves, stems, and sepals, and grayscale value. (**E**). Accumulation analysis of pFT:GUS in Atarf6/pFT:GUS, WT/pFT:GUS, and ZmARF6/pFT:GUS transgenic *Arabidopsis*. (**F**). *ZmARF16* was transiently transformed into *maize* protoplasts. The expression vectors for GFP and the fusion protein of ZmARF16:GFP were co-transformed with an expression vector containing a nuclear localization signal (NLS) fused to red fluorescent protein (NLS:RFP )nuclear marker)) in *maize* mesophyll protoplasts. GFP, nuclear marker, and chloroplast autofluorescence signals are individually labeled in green, red, and blue, respectively. Scale bar = 25 μm. (**G**) Expression of *ZCN12* in *maize* protoplasts and Western blotting analysis of florigen. (**H**) Expression of *ZmARF16* in *maize* protoplasts in 20μM IAA. *n* = 5. * *p* < 0.05, ** *p* < 0.01.

In order to understand how *ZmARF16* controls flowering time, we next compared the expression patterns of different flowering-related genes in *ZmARF16*–OX1, *ZmARF16*–OX2, WT, *arf6*–*1*, and *arf6*–*2* plants. Interestingly, several key flowering-related genes, including *AtSOC1*, *AtFT* and *AtAP1*, showed lower expressions in *arf6* plants compared to the wild-type plants, but higher expressions in *ZmARF16* overexpression plants (Figure 5A–C, Figure S4). We introduced pAtFT:GUS into ZmARF16–OX and *arf6* plants through hybridization, and found that GUS mainly accumulated in the leaf veins, similarly to the *ZmARF16* accumulation pattern. GUS accumulation was mostly seen in the leaves and calyx, and was least notable in the stem. The GUS activity in *ZmARF16*–OX was significantly higher than that in the wild type, while the GUS activity in arf6 plants was lower than that in the wild type (Figure 5E).

Subsequently, through Western blotting analysis (Figure 5D), we found that the highest degree of accumulation of florigen was in the leaves and calyx of *ZmARF16*–OX, and the lowest was in the stem. The expression level of florigen in *arf6* plants was lower than that in the wild type. These results indicate that *ZmARF16* may promote the initiation of flowering by positively regulating the expression of these flowering-related genes, as well as the accumulation of florigen.

To investigate whether or not *ZmARF16* enhances the transcriptional activity and florigen content of *ZCN12* in vivo (the steps for extracting protoplasts can be found in https://doi.org/10.1038/nprot.2007.199), we constructed ZmARF16–GFP and introduced it into *maize* protoplasts (Figure 5F). Utilizing GFP fluorescence, we selected protoplasts successfully transformed with ZmARF16–GFP. The overexpression of *ZmARF16* in *maize* protoplasts led to a significant increase in the content of *ZCN12* mRNA, as evidenced by quantitative measurements. Additionally, immunoblot analysis revealed an elevated level of florigen content in *maize* protoplasts overexpressing *ZmARF16* (Figure 5G). Consistently with the findings related to *Arabidopsis* transformation, *ZmARF16* demonstrated the ability to promote the expression of the *ZCN12* gene and increase florigen content in *maize* protoplasts (Figure 5H). Furthermore, when an elevated concentration of auxin was supplemented into the *maize* protoplast culture medium, a corresponding increase in *ZmARF16* mRNA content was observed.

### 2.7. ZmARF16 Positively Regulates ZCN12 and AtFT

The flowering integrator gene FT in *Arabidopsis* thaliana plays a crucial role in regulating the flowering process by activating the floral meristem. The expression of the AtFT gene was significantly increased in the plants overexpressing *ZmARF16*, and significantly decreased in the arf6 plants. A high degree of similarity was found in the amino acid sequence and protein tertiary structure between ZCN12 and AtFT (Figure 1B). Furthermore, both *ZCN12* and *AtFT* gene promoters contain the core element AuxRE (TGTC) (Figure S5), and they show similar functions and responses in the flowering process.

**Figure 6:**
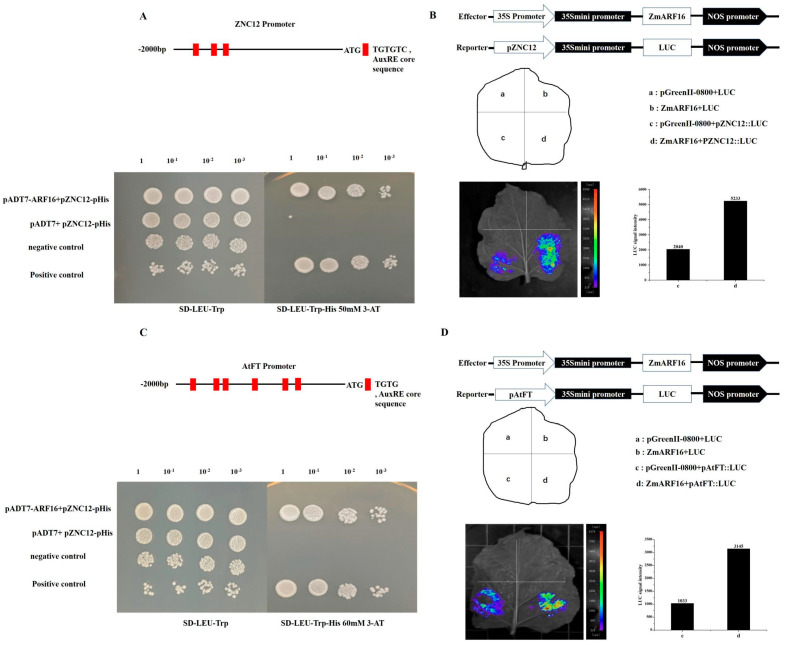
ZmARF16 accelerates flowering by promoting the transcription of *ZCN12* and *AtFT*. (**A**) *ZCN12* promoter structural graphic and yeast one–hybrid (Y1H) experiment showing in vitro binding of ZmARF16 to the promoter fragment of *ZCN12*. (**B**) Structural graphic of 35s:ZmARF16 and pZCN12:LUC vector and graphic of injection location. Co–infiltration of pZmARF16 effector gene and pZCN12 reporter gene into tobacco leaves, and measurement of luciferase signal. The experiment shows that ZmARF16 upregulates the expression of *ZCN12*. (**C**) *ZCN12* promoter structural graphic and yeast one-hybrid (Y1H) experiment showing in vitro binding of ZmARF16 to the promoter fragment of AtFT. (**D**) Structural graphic of 35s:ZmARF16 and pAtFT:LUC vector and graphic of injection location. pZmARF16 effector gene and pAtFT reporter gene co-infiltrated into tobacco leaves, and the luciferase signal was measured. The experiment shows that *ZmARF16* upregulates the expression of *AtFT*.

To verify whether ZmARF16 binds to the *ZCN12* and *AtFT* promoters, we performed yeast one-hybrid (Y1H) analysis and dual-luciferase reporter gene analysis. The detection of Y1H shows that ZmARF16 binds to *ZCN12* and *AtFT* promoters in vitro. Then, the *ZCN12* and *AtFT* promoters were inserted into the pRI-mini35S-LUC vector (luciferase reporter vector) as reporters, and ZmARF16 driven by the 35S promoter was used as the effector in the tobacco leaf transient expression experiment. We observed that ZmARF16 binds to the *ZCN12* and *AtFT* promoters in vivo (Figure 6A,C). Both the Y1H and dual-luciferase assay analyses showed that ZmARF16 directly binds to the *ZCN12* and *AtFT* promoters, and activates the transcription of the corresponding genes (Figure 6B,D).

## 3. Discussion

In 1865, Julius von Sachs revealed through partial shading experiments performed on leaves of *Tropaeolum ntajus* and *Pharbitis purpurea* (L.) Voigt that flower-related substances could be produced in the leaves under light [15]. However, it was not until 1920, when Garner and Allard discovered the photoperiod phenomenon, that Julius’ research findings were recognized [16]. The plant FT gene family is highly conserved in evolution, and acts on various biological regulatory pathways. The gene encodes a small, mobile protein that is concentrated in leaf veins [17]. Corbesier and Tamaki’s research groups, respectively, discovered the FT protein and Hd3a protein (product of FT homolog Heading date 3a) in *Arabidopsis* and rice, which can be transported over long distances from the leaves to the tops of stems, and confirmed that these proteins are florigens [18,19]. Kobayashi’s research showed that there are four transcription factors downstream of florigen in rice, namely *OsMADS14*, *OsMADS15*, *OsMADS18*, and *PAP2/OsMADS34*, the first three belonging to the *AP1* (APETALA1)/*FUL* (FRUITFUL) evolutionary branch and the latter belonging to the SEPALLATA evolutionary branch. During the transition from vegetative to reproductive growth in the apical meristem, the expressions of these transcription factors are upregulated [20]. The genes that have been proven to affect *maize* flowering mainly include *ZmCCT* and *ZCN8* [21,22]. *ZmCCT* is a *maize* homolog of Ghd7 and the characteristic locus associated with *maize* flowering time. It can inhibit the flowering gene *ZCN8* to delay *maize* flowering [23]. *ZmCCT* is a key gene that affects the adaptation of *maize* from tropical to temperate regions [24]. It was found that three CCT domain genes, *ZmCCT9*, *ZmCCT10*, and *ZmCOL3*, play important roles in the photoperiod and circadian pathways in *maize* [25]. The researchers inhibited flowering under LD conditions by transducing photoperiod signals. Independent transposon insertions on two related *ZmCCT* genes can reduce the sensitivity of *maize* flowering to the photoperiod [24,25]. *ZCN8* is transcribed and translated in leaves, and then moves through the phloem to SAM, where it interacts with the DLF1 protein to promote the transition from nutritional development to reproductive development [26]. The *ZCN8* gene has demonstrated a flowering function, and has recently been shown to help with adaptation to flowering in high-latitude regions [27]. In this experiment, *ZCN12*, with high homology to *ZCN8*, was discovered, and it was verified that *ZCN12* has the function of promoting flowering, which provides a reference for exploring the functions of the *maize* PEBP family.

The transcription factor ARF integrates auxin signals, and is shown to play important roles in various stages of plant development, including embryogenesis [28], cotyledon development [29], the proper formation of vascular tissue [30], hypocotyl elongation [31], the maintenance of apical meristem [32], lateral root formation, fruit maturation, phototropism, and gravitropism [33]. In *Arabidopsis*, *AtARF6* is regulated by red and blue light, which regulates hypocotyl elongation [34]. *AtARF6* can also respond to ABA signals, and its ubiquitination increases and accelerates in response to ABA treatment [35]. Therefore, *AtARF6* is an important integrator of various plant hormone signals and regulates plant development and responses to environmental signals. Previous research has shown that *miR167d-ARF6* plays a crucial role in regulating flowering and stigma size [36]. In this study, the arf6 single mutant showed delayed flowering, as shown in Figure S3B. The delayed development of the stigma and reduced cell volume in the central cell cluster led to delayed stamen development, resulting in the closed flower morphology shown in Figure 3C. Prior to this, Guilfoyie’s research found that knocking out *arf6* and *arf8* delayed stamen development and reduced reproductive capacity, and the expression levels of *ARF6* and *ARF8* genes affected the time of flower maturation [37]. At the same time, *ARF6* and *ARF8* can promote hypocotyl and xylem expansion, and *ARF6* and *ARF8* can respond to gibberellin signaling and interact with the DELLA protein to regulate increased xylem and fiber yields [38]. Research has found that *CRY1* and *PHYB* can directly interact with *ARF6*, inhibit auxin signaling, and promote hypocotyl elongation under blue and red light conditions, respectively [39]. This is consistent with our research showing that *ZmARF16* can promote the hypocotyl. However, its potential role in flowering remains to be explored. In this study, we found that *ZmARF16* plays an important role in the initiation of flowering by directly regulating the expression of the main integrators of the flowering induction pathway, *ZCN12* and *AtFT*. Our work elaborated on the mechanism of *ZmARF16* involvement. We verified that *ZCN12* promotes flowering, and discovered that *ZmARF16* can promote flowering in transgenic *Arabidopsis* by analyzing the yeast one-hybrid and F-luciferase assays and confirming that *ZmARF16* can increase the gene expression of *ZCN12* and *AtFT*. These results suggest that *ZmARF16* is involved in the flowering process in *maize* and *Arabidopsis*.

Reports on the effects of auxin signal regulation on plant flowering are rare. A previous study reported that the *ARF4* gene in strawberries promotes flowering by binding to the *AP1* and *FUL* promoter and regulating gene expression [40]. In this study, it was also found that *ZmARF16* binds to the AuxRE element in the *ZCN12* promoter to participate in the flowering process. This evidence suggests that auxin signals play a critical role in plant flowering processes. According to previous research, the ARF family not only responds to Auxin signals, but also responds to other hormone signals, including *ERF72* and *ARF6*, which mediate embryonic axis elongation via their interaction [41]; DELLA, which responds to redox signals and binds with *ARF6* to control Fruit Initiation in Tomato [42]; and *OsARF4*, which responds to brassinolide, regulating rice leaf inclination [43]. These results indicate that different hormones can participate in plant development through ARF family genes. Although the roles of other hormones in plant flowering processes are unclear, they can be explored through ARF family genes as a bridge. Uncovering the roles of ARF family genes in flower regulation is an important condition for revealing the auxin and related hormone regulation networks in plant development.

Our findings reveal that *ZmARF16* can also promote the development of floral meristematic tissues, leading to an increase in cell numbers and inflorescence development. In the shoot apical meristem (SAM), analysis based on ProDR5:VENUS reporter expression has shown that the maximum auxin activity occurs at the origin of the organ primordium [44,45]. The growth hormone induces cell differentiation and organ growth, making it crucial for SAM development. Previous studies have indicated that ARF5/MONOPTERROS is crucial for the initiation of leaves and the formation of leaf venation, as well as the formation and maintenance of SAM [46,47]. *ARF5* suppresses the expressions of *ARR7* and *ARR15* in the central and peripheral regions of the meristematic tissue [48], indicating that *ARF5* integrates cytophorin and Auxin signals to maintain SAM. Zhang et al. found that the growth hormone promotes the expression of *ARF3* to inhibit cell division factor activity and achieve cell division termination [49]. *ARF2* and *PIF5* (as well as *PIF4*) interact directly to promote plant aging, and they share common target genes such as *ORESARA 1* (ORE1) and *STAY-GREEN 1* (SGR1), key aging-promoting genes in the ABS3-mediated aging pathway [50]. This is in contrast to the role played by ZmARF6 in this study, which is related to the activation/inhibition of transcription factors [51], highlighting the interlocking and complexity of the ARF gene family in response to growth hormone signals. Unraveling the role of *ZmARF16* in cell division will help to establish the auxin regulation network

In conclusion, our research results reveal that *ZmARF16* promotes *ZCN12* expression and participates in the *maize* flowering process under the mediation of auxin signaling. Additionally, *ZmARF16* can also regulate AtFT to promote flowering in *Arabidopsis*, suggesting that the role of auxin in regulating plant flowering is relatively conserved. The discovery of this mechanism contributes to breaking the balance between the growth period and the yield of *maize*, as well as resolving the problem of the mismatch between male and female *maize* during domestication. These findings enhance our understanding of the regulation of plant growth and development by auxin signaling.

## 4. Materials and Methods

### 4.1. Plant Materials

In this study, the wild type was taken as the Columbia ecotype of *Arabidopsis* thaliana, and all mutant or overexpression lines were generated on the wild-type background. The 35s:ZmARF16 strains (*ZmARF16*–OX1 and *ZmARF16*–OX2) were sourced from Jilin Changchun (Jilin Agricultural University). The arf6 and ft mutants were purchased from ABRC. The 35s:ZCN12/ft line was generated through hybridization of 35s:ZCN12 with ft. The 35S:ZmARF16 vector was constructed and introduced into an Agrobacterium. Using Agrobacterium-mediated transformation, the vector was transferred into *maize* callus tissue, and transgenic plants were selected. The primers used in this study are listed in Table S1.

Atrf6 (CS24606) and Atft (CS9869) *Arabidopsis* mutants of ABRC

### 4.2. Comparison of Protein Structures

We import the amino acid sequences of ZCN12, ZCN8, and AtFT into DNAMAN(5.0 Version) for analysis, followed by color modification and layout adjustment.

We obtained the protein PDB files of AtFT and ZCN12 from NCBI, and then opened the PDB files with PyMol (3.0 Version) to obtain the protein tertiary structures of AtFT and ZCN12. Then, we performed structural overlap alignment of the protein tertiary structures of AtFT and ZCN12 to obtain RMSD values. The smaller the value, the more similar the protein tertiary structures were.

### 4.3. Flowering Time Determination

To determine flowering time, various plant materials were grown under day-long (LD: 16 h light/8 h dark) conditions at 22 °C, and the flowering time was measured. The average value of 20 plants was taken for each measurement. *Maize* grew under field conditions.

### 4.4. Subcellular Localization

LA Taq polymerase (Trizol reagent, Changchun, China) was used. We amplified the CDS fragments of primers *ZmARF16* and *ZCN12*. PCR was performed under the following conditions: 95 °C for 5 min, followed by 35 cycles of 95 °C for 30 s, 58 °C for 30 s, 72 °C for 10 min, and finally extension at 72 °C for 5 min. Then, we constructed 35S:*ZmARF16*-GFP and 35S:*ZCN12*-GFP vectors. The pCAMBIA1302-GFP vector containing 35S:GFP (GFP) was used as a control, and it was transferred into Agrobacterium GV3101. Subsequently, the bacterial solution was injected into tobacco leaves and *maize* protoplasts. Two days later, the GFP fluorescence was imaged using a confocal laser scanning fluorescence microscope (TCS SP8-SE; Leica, Wetzlar, Germany) with an excitation wavelength of 488 nm.

### 4.5. Auxin Treatment

In order to determine the relative gene expression, 2-week-old seedlings (2-week-old seedlings) grown on 1/2 MS culture medium were transferred to 1/2 MS culture medium with the addition of 15 μM IAA and cultivated for 12 days, measuring the length of the hypocotyl. Then, normal 20-day-old wild type *Arabidopsis* leaves were sprayed with water and different concentrations of IAA, i.e., 10 μM, 15 μM, 20 μM, and gene expression was analyzed by harvesting the leaves one hour later.

### 4.6. Gene Quantitative Analysis

Total RNA was extracted from the seedlings using TRIzol reagent (Invitrogen, Waltham, MA, USA). The total RNA (1 μg) was pre-treated with DNase I and reverse-transcribed with Superscript II (Invitrogen), as shown in Tables S2 and S3. Quantitative reverse transcription-polymerase chain reaction was performed using a real-time fluorescence quantitative PCR machine to determine the expression of the genes. The assay was repeated three times. Subsequently, we calculated the 2^−∆∆Ct^ value to analyze the relative gene expression. ACTIN2 was used as an internal control, and the gene-specific primers are listed in Table S1.

### 4.7. GUS Staining

We perform GUS activity staining on 10-day-old seedlings of pZmARF16: GUS or pAtFT: GUS transgenic plants. We placed the material in 90% acetone and treated it at room temperature for 20 min. We removed the acetone, added GUS staining solution ((100 mmol/L sodium phosphate buffer (pH = 7.0), containing 0.5 mg/mL X-Gluc, 1% Triton X-100, 1% DMSO, and 10 mmol/L EDTA)), and stained the material in the dark until a signal appeared. After the reaction was complete, we rinsed the seedlings twice with 70% (*v*/*v*) ethanol to remove chlorophyll and take tissue images (Canon EOS R5).

### 4.8. Yeast One Hybridization Test

The *ZmARF16* CDS sequence was linked to the pGADT7 vector. The promoter sequences (2000 bp) of *ZCN12* and *AtFT* were cloned and analyzed to determine the presence of AuxREs. The promoter of *ZCN12* AuxRE and the AtFT promoter were amplified and inserted into the pHis2 vector. The primers are listed in Table S1. The vectors containing ZmARF16 and the *ZCN12* and *AtFT* promoter fragments were co-transformed into Y187. Transformed yeast strains were grown on SD/–Trp medium and selected on SD/–Trp–His–Ade medium(Coolaber, Beijing, China). The interaction analysis was conducted by observing yeast growth.

### 4.9. Transcriptional Activity Analysis

BD-ZmARF16 and the empty vector pGBDT7 (BD) were then transformed into yeast Y187 (Saccharomyces cerevisiae) for transcriptional activity analysis. The transformed yeast strains were grown on SD/–Trp medium(Coolaber, Beijing) and selected on SD/–Trp–His–Ade medium. The transcriptional activity of *ZmARF16* was determined by observing yeast growth.

### 4.10. The Dual-Luciferase Assay

We conducted real-time expression analysis using Nicotiana benthamiana leaves. To generate the desired constructions, the 2 kb *AtFT* promoter and *ZCN12* promoter were cloned into the pGreenII 0800-LUC vector, producing *AtFT*pro:LUC (reporter gene construct) and *ZCN12*pro:LUC, respectively. The sequence of *ZmARF16* was inserted into the pGreenII 62-SK vector, generating 35S:ZmARF16 (effector gene construct). Nicotiana benthamiana plants grew 7–8 leaves, and 3–4 middle leaves were selected for the experiment. The reporter and effector genes were transformed into the Agrobacterium strain GV3101 and co-infiltrated into Nicotiana benthamiana leaves. After normal grow for 48–72 h, we prepared a D-Luciferin potassium salt solution (150 μg/mL) and sprayed it onto the tobacco leaves. Five minutes later, we used a low-light cooled CCD imaging(AniView100, Guangzhou, China) device to expose them for 5 min, 10 min, and 20 min, and measured the light emission intensity. The real-time expression experiments were performed with three independent biological replicates.

### 4.11. Toloniumchloride Staining

We placed the sample in a 50% FAA fixative solution for 12 h (the volume ratio of fixative solution to tissue block was 10:1). The sample was stained with 0.5% chlorotoluene solution for 30 min, and the excess dye was washed away with water. We soaked the sample in 95% ethanol for 1 min, dehydrated it with xylene, and then embedded it in paraffin. After slicing, the tissue was observed under an optical microscope, and the image was saved after determining the field of view (Olympus, Tokyo, Japan, BX53).

### 4.12. Western Blotting

From the *ZmARF16*-OX and *arf6 Arabidopsis* plant, samples, leaves, stems, and calyx tissues were collected. Total leaf protein was extracted using 0.5 mL of plant extraction buffer (CWBIO, Beijing, China), and the supernatant was used as the input sample after centrifugation at 12,000 g for 20 min. We conducted the total protein SDS experiment, and transferred the protein onto a PVDF membrane. Then, we added the flowering-specific antibody PHY0028A (1:1000) and incubated it for 2–3 h. After that, we added the secondary antibody (Horseradish Peroxidase 1:2000) and incubated it for 1–2 h. We washed it with TBSTB buffer, and then proceeded with exposure. We saved and retained the images(AniView100, Guangzhou).

### 4.13. Preparation of Maize Protoplasts and Transform

We cut the two ends of the *maize* leaves into 0.5~1.0 mm sections, placed them into the enzymatic solution, and shook it at room temperature in the dark (rotation speed of 40 r/min) for 2–7 h for enzymatic hydrolysis. Before removing undissolved leaves, we used an equal amount of 2 mmol/L MES (pH 5.7). We diluted the enzyme solution containing protoplasts with 154 mmol/L NaCl, 125 mmol/L CaCl_2_, and 5 mmol/L KCl solution. We filtered the enzymatic hydrolysate containing protoplasts through a sieve. The conditions were as follows: 400 r/min, 2 min, one repeat, an d supernatant removal. We added an appropriate amount of 4 mmol/L MES, 0 4 mmol/L D-mannitol, and 15 mmol/L MgCl_2_, and set the material aside.

We placed 200 μL of *maize* protoplasts into a 2 mL centrifuge tube and added 10 μg of the 35S: ZmARF16-GFP plasmid, mixing the contents well. We let them sit on ice for 30 min, then put them under heat shock at 45 °C for 5 min, followed by placing them for 2 min on ice. We incubated them in the dark at room temperature for 30 min. We added an equal volume of PEG/Mg^2+^ solution (100 g/L PEG 4000 and 50 g/L magnesium chloride) and incubated them in the dark for 10 min. Then, we added 4 mL of washing solution (30 g/L KCl, 5 g/L CaCl_2_·2H_2_O and 36 g/L mannitol, pH 5.6) and induced the reaction under low light at room temperature for 16 h. We centrifuged the material at 2000 r/min for 5 min at 4 °C, discarded the supernatant, resuspended the protoplasts in 200 μL of washing solution, and observed them under a fluorescenceinverted microscope(TCS SP8-SE; Leica, Germany).

## Data Availability

The original contributions presented in the study are included in the article/Supplementary Material, further inquiries can be directed to the corresponding author.

## Supplementary Materials

The following supporting information can be downloaded at https://www.mdpi.com/article/10.3390/ijms25179607/s1.
