# Supplementary material for: ZmARF16 Regulates ZCN12 to Promote the Accumulation of Florigen and Accelerate Flowering
Source: Int J Mol Sci. 2024 Sep 5;25(17):9607. doi: 10.3390/ijms25179607 (PMC11395262; doi:10.3390/ijms25179607)
Supplement: Supplementary file 1 [file ijms-25-09607-s001.zip › ijms-3138152-supplementary.pdf]

ZmARF16 regulates ZCN12 to promote the accumulation of florigen and accelerate flowering

Supplementary

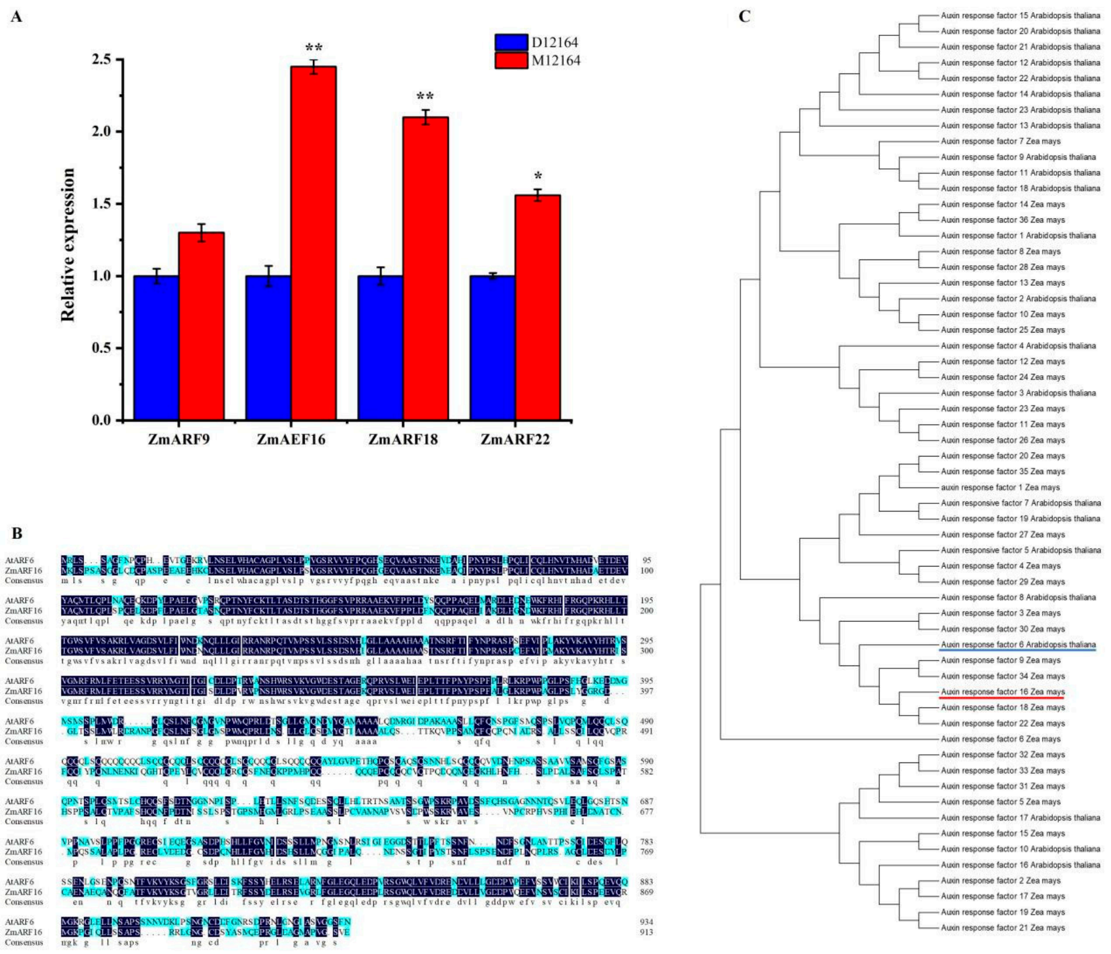

Figure S1. A.ZmARF9, ZmARF16, ZmARF18, and ZmARF22 expression level in D12164 and M12164. B–C. Homologous proteins and sequence alignment of the ZmARF16 gene.

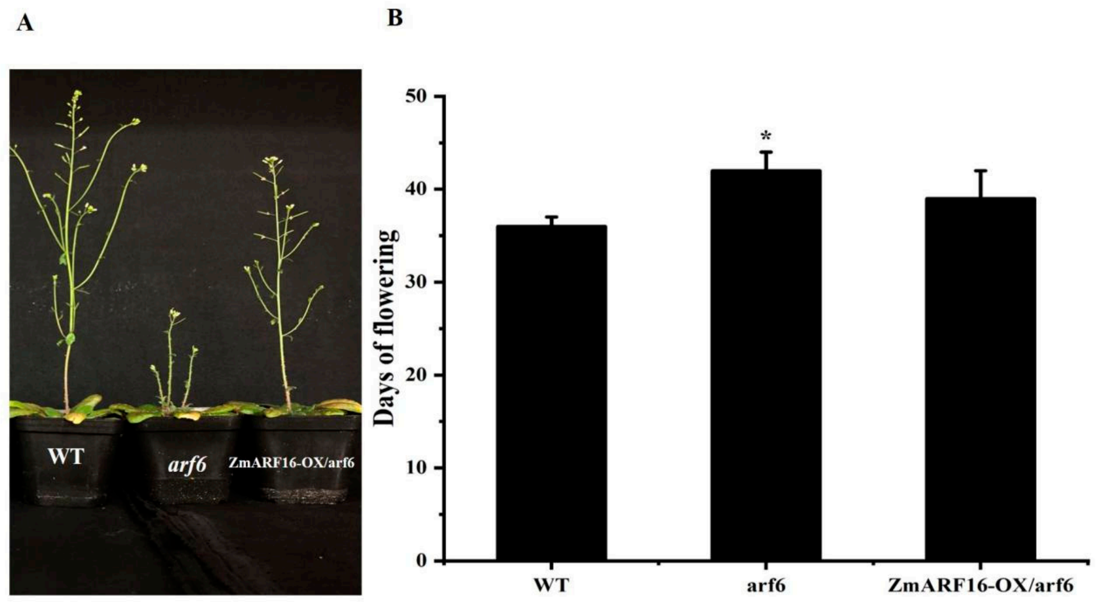

Figure S2. ZmARF16 can alleviate the early flowering phenotype caused by arf6 gene mutation . A. Phenotypes of WT, arf6, and ZmARF16-OX/arf6 plants under long-day conditions. B. Flowering time of WT, arf6, and ZmARF16-OX/arf6 plants under long-day conditions.

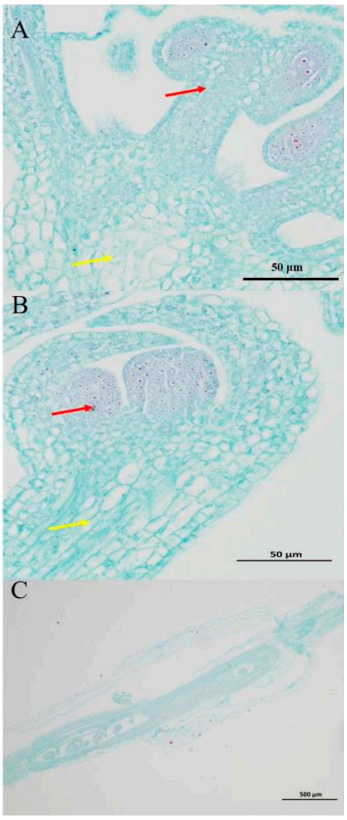

Figure S3. Observation of floral meristem in WT, arf6, and ZmARF16-OX lines at 35 days. A shows the pod structure of WT. B and C show floral meristem of arf6 and ZmARF16-OX plants. Red arrow: stigma. Yellow arrow: central meristem.

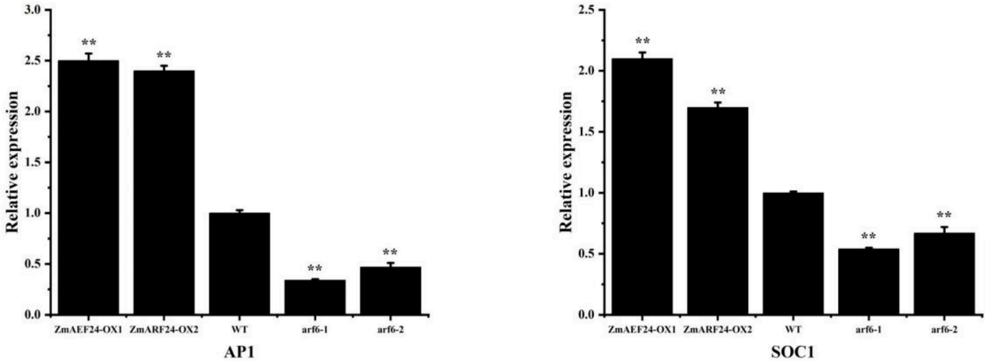

Figure S4. Expression levels of AP1 and SOC1 genes in leaves, stems, and calyxes. **: $p<0.01$.

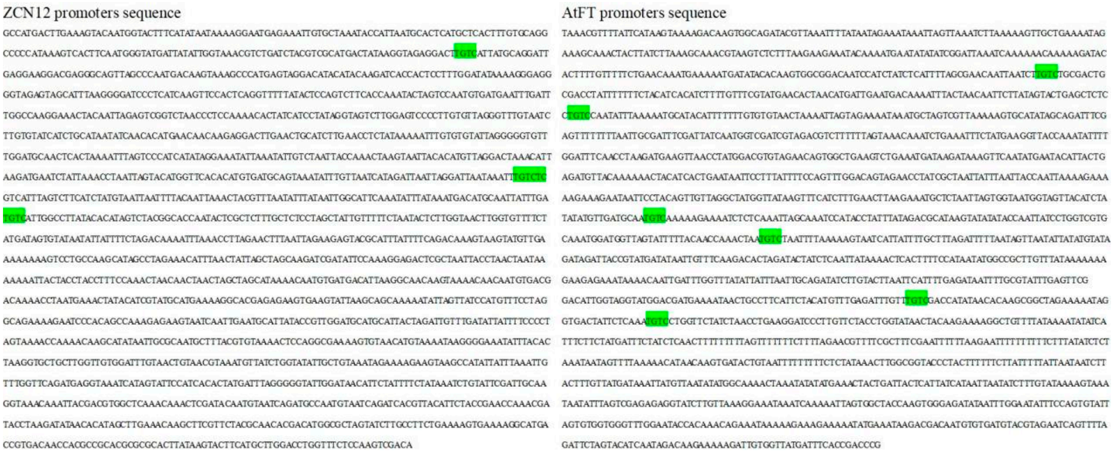

Figure S5. Upstream 2000bp promoter sequences of ZCN12 and AtFT genes. The green label denotes the core element AuxRE.

Table S1.Primer sequence

name	sequence（5’-3’）
AtFT-F	TCACCACTCCAGTCCATGAC
AtFT-R	TATTACCGGCTCCAGAGTGC
ZmARF16-F	CTGTGGCCACCAAAAGTGTG
ZmARF16-R	TGAACCTCGAACCAGCAGAA
AtAP1-F	CTTTTGAAGAAAGCTCATGA
AtAP1-R	TGTTGCTCTTGTTGTCTTCT
AtSOC1-F	ATGGTTTGTTGAAGAAAGCCTT
AtSOC1-R	TTGGGCTACTCTCTTCATCAC
ZCN12-F	TTGTGAATAAGCCAAGAGT
ZCN12-R	TGTACCTCGGCTCTGGCCTC
Actin2-F	TGGAGAAGATGACCCAGATC
Actin2-R	TCTGAAGAACTTGTTCCAA
ZmARF9-F	CGGAGCCACATGAGAGAAGAA
ZmARF9-R	AAGCTAGATACTTACCAGCAGATGG
ZmARF18-F	TGAGCCTGAGCGGAAAAG
ZmARF18-R	ACAAAGCAGGCATCGCCCACAATG
ZmARF22-F	GCCACCGAACAGAATCAAGA
ZmARF22-R	TTCCTCAAACAGCAGATCCAA

Table S2.Reverse transcription system

Component	Volume
SureScript RTase Mix(20×)	1μL
SureScript RT Reaction Buffer(5×)	4μL
Total RNA	1μL
ddH_2O	Add to 20μL
Total	20μL

Table S3.Reverse transcription Procedures

Temperature	Time
25℃	5 min
42℃	15 min
85℃	5 min
4℃	hold
